# Nursing Students' Perception of Clinical Teaching and Learning in Ghana: A Descriptive Qualitative Study

**DOI:** 10.1155/2022/7222196

**Published:** 2022-04-18

**Authors:** Sarah Ama Amoo, Yaa Boahemaa Gyasi Aderoju, Richard Sarfo-Walters, Patience Fakornam Doe, Christiana Okantey, Christian Makafui Boso, Susanna Aba Abraham, Andrews Adjei Druye, Nancy Innocentia Ebu Enyan

**Affiliations:** ^1^Cape Coast Teaching Hospital, Intensive Care Unit, Cape Coast, Ghana; ^2^University of Cape Coast, School of Nursing and Midwifery, Cape Coast, Ghana

## Abstract

**Background:**

Clinical teaching and learning are critical in bridging the theory-practice gap in nursing education. This study aimed at exploring nursing students' perception of clinical teaching and learning in Ghana. In particular, this study sought to (1) describe the factors that promote clinical teaching, (2) examine students' perception of clinical teaching, (3) describe the impact of clinical learning on students, and (4) explore ways of improving clinical teaching and learning.

**Methods:**

A descriptive qualitative study was conducted with 16 final-year nursing students using telephone-based interviews. Individual in-depth interviews were conducted with a semistructured interview guide, and data were analysed by the qualitative thematic analysis.

**Results:**

The findings indicate that being taught new things, being supervised, and having autonomy were the most significant factors that promoted clinical learning. Participants also reported that clinical experience created learning opportunities that helped develop clinical competence. They described learning experiences in the clinical setting as good, albeit gaps in practice. Poor staff attitude, lack of equipment, poor student attitude, inadequate learning opportunities, and lack of clinical supervisors were perceived as challenges in the clinical environment.

**Conclusions:**

Efforts to consciously teach, supervise, and challenge students to have independence in the clinical area will promote clinical teaching and learning. Therefore, nursing educational institutions and all other stakeholders need to collaborate in eliminating the numerous challenges students encounter in the clinical environment.

## 1. Background

Clinical teaching and learning are integral components of nursing education. Most clinical teaching and learning activities occur in the clinical setting where theory is translated into practice [1, 2] in healthcare providing institutions [3, 4]. Although the clinical environment is a significant place to apply the theories learned in the classroom, there remains a gap in the magnitude of applying the theory to practice. The effective application of theory to practice depends on many factors, including creating learning opportunities for students and support by preceptors [5].

A conducive clinical environment is critical in enhancing the learning experiences of students. For instance, in an environment where simulation learning is limited or not available at all, learning occurs in the actual hospital environment [6]. However, most clinical training facilities in sub-Saharan Africa are constrained by logistics and equipment, which tends to affect students' learning experiences. Multiple factors have been reported to affect student learning in the clinical area. These include individual-level factors, the nature of the hospital environment, socioeconomic, and nurse educator factors [7]. A study conducted by Rajeswaran [8] in Botswana discovered that nursing students could not translate the theory into practice because they lacked adequate supervision in the clinical setting, which resulted in low performance in clinical practice.

Research has identified several factors that negatively impact the clinical performance of students. These included limited opportunities for students to practice in teaching hospitals, inadequate or nonavailability of nurse educators, clinical instructors and mentors, and too many students in the programme [9]. It has also been reported that clinical instructors' attitude, constructive criticism, and supportive clinical settings promote clinical learning. Nonetheless, negative criticism tends to affect students' clinical performance [1]. The clinical teaching and learning process can also be negatively affected by student-related factors such as inadequate knowledge and skills, poor attitude, unprofessional behaviour, and poor communication skills with patients and clinical instructors. Again, the inability of students to ask questions, overconfidence, not being motivated to learn or work, lack of confidence, and dishonesty have been cited in the literature [10].

Despite the scarcity of evidence on students' perception regarding clinical education in Ghana, other studies globally reported nursing students' education and professional socialising experiences, processes, and outcomes are influenced by the quality of clinical learning setting and their perception of it during clinical placements [11, 12]. It has been revealed that nursing students perceived the clinical setting as stressful and challenging attitude [13]. However, others perceived the clinical education experiences as rewarding and satisfying [14].

Generally, from the students' perspective, the clinical setting has been described as nonsupportive because of institutional inadequacies, a lack of relationship between students and clinical educators, and negative attitude and behaviours on the part of some nurse educators [15]. Students have highlighted inhibitors to clinical learning, including preceptors' inadequate engagement and feedback [16]. Moreover, preceptors were not available to engage with students. Students found that theory and practice were not connected and felt that they lacked opportunities to reflect together with their preceptors [17].

It is critical to note that clinical training in nursing occurs in a complex clinical learning setting, which is influenced by many factors [18]. This setting provides an advantage for student nurses to learn experimentally and to translate theoretical knowledge to a diversity of mental, psychological, and psychomotor skills, which are of importance for patient care [19]. Students' exposure and preparation to enter the clinical environment are significant factors influencing the quality of clinical education [20].

According to the Nursing and Midwifery Council (N&MC) of Ghana, the minimum practical hours expected on a Registered General Nursing Programme are 1632 hours in a minimum of 3 years. For the institutions that run 4-year Bachelor of Science in Nursing Programmes, most practical sessions begin in the second year when students are deemed to have acquired some professional knowledge and are adequately prepared at least for basic nursing procedures. Clinical rotation is designed to cover all specialty areas required by the N&MC, including medical/surgical nursing, obstetrics and gynaecological nursing, public health, mental health, paediatrics, and other selected specialties available in the specialist units.

Students are taught mainly by clinical faculty and preceptors in the clinical sites. Faculty from the schools also have specific days they visit the clinical sites to teach their students. The minimum qualification for faculty from the schools is Masters' degree. On the other hand, clinical faculty/preceptors are Masters or Bachelor of Science prepared nurses. The minimum qualification for professional nurses in Ghana is diploma. The assessment of students' clinical competencies is usually conducted at the end of each semester in the clinical sites or the schools' skill laboratories using standardised evaluation tools. These examinations are conducted by faculty from the schools and clinical faculty/preceptors and are most often graded according to the schools' grading systems.

In Ghana, improving clinical nursing education is a significant characteristic of clinical skill acquisition. Critical areas of consideration include improving a positive clinical environment, effective clinical supervision, adequate assessment of students, and clinical-academic collaborations [21]. This research is critical in improving clinical teaching and learning in the Cape Coast Metropolis of Ghana. Currently, there is a paucity of data regarding this topic in the study area. This study explored nursing students' perception of clinical teaching and learning, in particular to (1) describe the factors that promote clinical teaching, (2) examine students' perceptions of clinical teaching, (3) describe the impact of clinical learning on students, and (4) explore ways of improving clinical teaching and learning.

## 2. Methods

### 2.1. Design and Participants

The study employed a qualitative explorative, descriptive design. The population comprised final-year student nurses who had enrolled on a four-year Bachelor of Science in Nursing programme in a public university in Ghana. Purposive sampling was used to recruit participants who were 18 years or older and had at least three years of clinical teaching and were therefore information-rich per the study's objectives.

### 2.2. Study Setting

The setting for this study comprised clinical placement sites across the country. Students enrolled in a Bachelor of Science in Nursing programme are mandated to have practical experience within the semester and during the inter-semester break. These students are allowed to select sites for clinical placement based on their location and the type of health facility that can help them accomplish the objectives of the clinical practicum. These sites represent different levels of health facilities in Ghana, including teaching and district hospitals. Some of the facilities are owned by the government, while others are private facilities. Although the facilities provide health services, the level of sophistication and specialised services differs across facilities. Although most of the facilities provided both general and specialised services, few concentrated on the provision of mental health services. The teaching hospitals focused on providing both general and specialised services and served as referral points for the district hospitals and other facilities in the periphery.

### 2.3. Data Collection

A semi-structured telephone-based interview technique was used for inquiry. The interviews were conducted between June and July 2020 to obtain in-depth information about participants' perception regarding clinical teaching and learning. It is worth noting that at the initial stages of the pandemic in March 2020, all clinical activities in the placement sites were halted. Students went home and continued classes online. Nonetheless, some of the students continued to support the staff in their various hospitals due to the staff shortages. They returned to campus to complete the semester in September 2020 after some restrictions had been lifted. At the time of the interviews, students were not on campus, which impacted our ability to do face-to-face interviews.

Demographic information comprising sex and age was collected prior to each interview to give perspective on the data. The semi-structured interview covered the following index questions followed with several probes: (1) How do you perceive the clinical teaching and learning environment? (2) How will you describe the clinical teaching you received? (3) What professional skills and attitudes did you model or learn from the professionals you worked with?

A total of sixteen interviews were conducted during the study period, at which point data saturation had been confirmed. The interviews were conducted in English and audio-recorded after consent had been sought from the participants. Each interview lasted between 45 and 60 minutes.

### 2.4. Data Management and Analysis

The inductive thematic analysis was conducted guided by the six steps proposed by Braun and Clarke [22]. Audio recordings were saved on separate password-protected computers, and the data were transcribed verbatim and analysed by two members of the research team. Each independent researcher read and reread the printed transcripts and listened to the audio version concurrently for familiarisation with the data. Afterwards, relevant phrases or sentences were highlighted and short codes were assigned.

The highlighted data were then collated into groups to get a condensed overview of the main points recurring throughout the participants' narratives. Following coding, the themes were generated by identifying patterns among the codes and combining several codes. After that, independent researchers deliberated and agreed on the themes to ensure accurate representation of the participants' narratives. The emergent themes were then defined by explaining exactly what the themes mean in relation to the data set.

### 2.5. Trustworthiness

Rigor was maintained throughout the research process as recommended by Lincoln and Guba [23]. To establish credibility, member checking was carried out. Also, two members of the research team analysed the data independently to ensure congruence between data and themes. An audit trail was also maintained to achieve confirmability and dependability by detailing the research process, the participants, and research setting. Two members of the research team coded the transcript. The audio-recorded interviews and transcripts were kept safe to allow for transferability.

## 3. Results

Sixteen final-year nursing students offering Bachelor of Science in Nursing in a public university were interviewed for the study.  Table 1 shows the demographic characteristics of the participants. Eleven of the participants were females, and five were males and were aged between 21 and 25 years. Five main themes and several subthemes emerged from the data, as shown in Table 2. The five themes are factors that promote clinical learning, the impact of clinical experience on students, students' perception of clinical teaching, challenges in the clinical setting, and improving clinical teaching and learning.

### 3.1. Factors That Promoted Clinical Learning

This theme described the many and varied factors that promoted clinical learning among students. Most of the participants were excited about an opportunity to learn new things in the clinical setting and described their best clinical experience as the one in which they were taught new things. They had the autonomy to practice and were supervised to practice new things learned on the ward. These factors that promote clinical learning are explicated as follows:

#### 3.1.1. Being Taught New Things

Most participants reported that being taught new things promoted clinical learning as indicated in the following quote:

*“… They taught us the emergency drugs. So that made me yearn for more. So, I mean, my best experience so far was at the Hospital A where I had nurses teaching me day by day. Teaching me the instruments, anything they had to do”* (Participant 1).

#### 3.1.2. Being Supervised

Participants indicated that being supervised facilitated learning in the clinical area:

“*…We were supposed to perform a procedure, I think I did not do it well, and then our clinical instructor, one of the sisters, actually approached me and then corrected me. I think that was the best because of the way she approached me and made me understand that we do not do this at this point and she did not shout at me, maybe that is why I feel it's the best, and she actually corrected me at her office not right in front of the patients”* (Participant 6).

#### 3.1.3. Having the Autonomy to Practice

Most of the participants described having the autonomy to practice as a factor that promotes clinical learning:

*“… I learned that you can do a lot of things for a patient, and then you get to have the opportunity to do things on your own. You are supervised, but then we have a certain autonomy. You get to know how things are done. [In] certain hospitals, you do not get to practice the skill itself but here you are given the opportunity to actually do…wound dressing… and all that”* (Participant 10).

### 3.2. The Impact of Clinical Experience on Students

Clinical practice plays an important role in helping shape students' clinical skills needed for professional nursing practice. The participants shared how the clinical practices impacted them. The subthemes are clinical learning opportunities and develop clinical competence.

#### 3.2.1. Clinical Learning Opportunity

Most participants described it as a learning opportunity.

*“We change hospitals almost every semester, so if you are at a certain hospital where you do not get to experience a lot of conditions, you get to experience it at another place”* (Participant 10).

*“Even though sometimes we are handicapped with the basic tools and equipment that we are supposed to practice with, at least we are able to learn one or two before you leave the ward”* (Participant).

#### 3.2.2. Developing Clinical Competence

Some participants shared how their opportunities in the clinical setting to practice have improved their clinical competence:

*“I did oral suctioning. That was the first time I actually did suctioning. I had observed on several occasions, but I never had the opportunity to do it. That was the first time I prepared the patient, used the suctioning machine myself, and inserted it. I was supervised for three times and subsequent ones I did them myself”* (Participant 11).

*“I have experienced so many clinical experiences, but the one I really appreciate is how to pass an IV line and serve medication. Initially, at the lower levels, I had been having difficulties, and thought I will not be able to, but with exposure, [I can] do it now. Initially, people normally help me and supervise but now I can do it”* (Participant 13).

### 3.3. Students' Perception of Clinical Teaching

Participants have had varied clinical teaching experiences and therefore perceived clinical teaching differently. While some described it as good, others taught there were gaps, and some said it was inadequate.

#### 3.3.1. Clinical Practice Is Good

In sharing their clinical experience, some participants described their clinical practice as good. This was especially the case when participants had the opportunity to learn new things and linked theory to practice examples:

*“… I think it's very good and the opportunity to go to hospital B too was very educative. I think it was very good even though some of us thought it was hectic, we were able to learn something”* (Participant 10).

*“Everything is good, especially the time we went to hospital B that one was very good” (Participant 13).*

*“… I will say that so far I have learned so many things and then although we are still learning what I have learned I can say that it is good for me”* (Participant 4).

#### 3.3.2. Gaps in Clinical Practice

Nearly all participants believed that there is a theory-practice gap. Some participants indicated that they struggled to link theory to practice as what they did in school was different from what they experienced at the clinical area. They identified inadequate time, lack of equipment, and inadequate learning opportunities as major contributing factors to the theory-practice gap:

“*… In theory or in class, we are told what we are supposed to do and what you are not supposed to do, but you go to the ward at times they will say time factor, so they do ‘short cut', and we miss some of the steps. And at times too, we do not have the required equipment, so we have to improvise. So, we get to the ward, and you see there is a big gap”* (Participant 7).

“*Sometimes you just do not see whatever is taught in the classroom on the ward and even on the practical aspect we learn [a] task that when we go to the ward, we do not see them at all”* (Participant 6).

#### 3.3.3. Inadequate Clinical Practice

Some participants perceived their clinical experience to be inadequate and, therefore, did not acquire the clinical experience they would had expected:

*“Comparing it to what we actually learn at the skills lab, I would say it's not adequate in the sense that we do not actually get the opportunity to practice everything that we've learned. What we do will sometimes be dependent on what is happening at the ward”* (Participant 8).

“*If you get to certain wards and you do not meet [ward] in-charges who are willing and always devoted to help students,…we only go there and be asked to do this, do that, go and do this and then by the time you realise your session has ended and you have to leave the ward to the school”* (Participant 9).

### 3.4. Challenges in the Clinical Setting

This subtheme describes perceived challenges to effective clinical teaching. Participants reported several students, staff, and clinical environmental factors. These included poor staff attitude, lack of equipment, poor student attitudes, lack of learning opportunities, and lack of supervisors.

#### 3.4.1. Poor Staff Attitude

Some participants bemoaned the negative attitude of staff nurses toward students. This attitude, they said, affected their ability to learn and achieve their fullest potential in the clinical setting:

*“There were times that you go to the ward and then you do something, the nurses shout at you. Sometimes they will not even mind you. Sometimes you even try to help, and then they are like, ‘go and sit down. I have to finish and document my thing and go'. Some of the attitudes sometimes are bad”* (Participant 6).

*“What has been our problem throughout this training session has been [the] attitude of most [the] staff and in-charges in the ward. Sometimes most of us go there, and we really want to get involved in what is going on and know better, but the response somebody might give out to you would not even encourage you to get closer to the person and know much from the person. That is the main reason why sometimes we go to the ward, and you see students loitering around the hospital only because we feel the behaviours of some staff and in charges are intimidating”* (Participant 9).

#### 3.4.2. Lack of Equipment

Lack of equipment prevented students from practicing what they had been taught in the classroom:

*“My challenge is that some of the clinical institutions do not have the equipment, so we have to improvise”* (Participant 5).

*“... I will use wound dressing, for example, we've been taught how to use the instrument for wound dressing and also how to use the sterile glove, but the issue at hand is for the three years, it's always the sterile glove that we use, there is no instrument for you to use. So, although we know its theory, we do not know it in practice”* (Participant 7).

#### 3.4.3. Poor Student Attitude

To some participants, students' own attitude also affected their learning in the clinical setting:

“*The other thing is the students' attitude too. Sometimes, the attitude of the students annoys the nurses, that is, why they are not able to teach us, or they are not willing to teach, so when we go to the ward, we should humble ourselves, we should respect them as they are”* (Participant 11).

“*The nurses and the midwives look at how you students behave before they impart knowledge to you. First of all, when you go to the clinical setting, and then you exhibit that kind of zeal for the work, they also help us to develop our confidence, but if we go there and we do not feel like doing the work, they do not mind us, so that is one aspect of it”* (Participant 5).

#### 3.4.4. Lack of Learning Opportunities

Although they are in the clinical setting to learn, the participants complained that they often felt left out and were not allowed to learn or practice the skills they are taught in school.

“*We went to different clinical facilities; some are very conducive, and some … are (sic) not. The ones that are conducive when the patient comes students are allowed to work with them, but in some hospitals, students are supposed to observe. Thus, we observe, we do not participate in anything we just observe”* (Participant 13).

“*The teaching of students has not really sunk down in some hospitals, so they feel students come and they are just there to volunteer and then learn through the process”* (Participant 3).

*“Sometimes they do not give students the opportunity to do certain procedures that we have been taught in the class”* (Participant 14).

#### 3.4.5. Lack of Supervisors

Participants complained about the lack of supervisors in some of the clinical setting which was illustrated by the following quote:

“*We do not have clinical instructors to even guide us to learn whatever we have studied in School. We just go to the clinical setting; we present the objectives to them, and we will be doing something outside what we are supposed to do”* (Participant 1).

### 3.5. Improving Clinical Teaching and Learning

The theme highlighted the factors that can improve clinical teaching and learning. The factors identified were supervision, training of staff, separate practical and theory sessions, and strengthening clinical teaching.

#### 3.5.1. Supervision

Most of the participants indicated that clinical supervision was key in improving clinical practice:

“...*There should be clinical supervisors or instructors in the clinical setting who will guide students in whatever they do*” (Participant 1).

“... *I wish we could have a setting whereby some of our lecturers will be with us on the ward, especially with medical-surgical, bridging those gaps. Sometimes you just do not see whatever is taught in the classroom on the ward and even with the clinical aspect, we learn [certain] task[s]… [but] when we go to the ward, we do not see them at all*” (Participant 6).

“...*In the medical field, they have supervisors that come around, and they will go around. I'm suggesting that if the nursing department can do that, it will help improve our learning in the field. Supervisors are around, we go through the patient's folders, after that we all sit down and try to learn about it*” (Participant 12).

#### 3.5.2. Training for Staff

Participants also highlighted the need for periodic training for clinical staff as illustrated in the quote below:

*“...They should be put through some training maybe every three months or every two months to refresh everything they know because you know nursing sometimes certain things change. So that when something new comes into the system, they will be able to get an idea about it so that they would not go through the old way repeatedly”* (Participant 5).

#### 3.5.3. Separate Practical and Theory Sessions

The participants highlighted the need for theory and practice sessions to bridge the theory to practice gap, as illustrated by the quote below:

“… *If we set a period aside, where we finish class session, allocate some specific time for the clinical session and then come back so that we get to know the strong linkage between the two separate trainings, we are receiving”* (Participant 9).

#### 3.5.4. Strengthening Clinical Teaching

The participants described measures for strengthening clinical teaching as illustrated by the following quotes:

“*... At times, we would not get the nurses having time because of their duty schedule, but if your tutors' come around and they have time for you, I think that one will facilitate teaching and learning much effectively”* (Participant 4).

“*...When students go to the ward, I suggest that after we are done, at least they should sit us down or gather us somewhere and lecture us on the ward. They should teach us new things. They are very good so they can sit us down and we can have some presentations and all that and not just with students for vital signs”* (Participant 14).

## 4. Discussion

This study aimed at exploring nursing students' perception of clinical teaching and learning in Ghana. The results revealed that students generally perceived clinical learning experience as an important requirement for achieving clinical competence. However, several factors are related to students, staff, and challenges within the clinical setting as barriers to effective clinical education. On the factors that promoted clinical learning, most of the students believed that maximum learning is attained when they are taught new things, properly supervised, and have adequate time and space to practice. In essence, the students valued much when they had mentorship or a chaperone to guide them through acquiring new knowledge and being supported to practice what is taught. It is evident that constructive feedback and resources are critical in promoting effective mentoring practices and bridging the theory-practice gap [Nkosi, 2017]. An earlier study found the nature of the mentoring relationship, quality of the mentor, and ability to facilitate learning, positive feedback, and timely decision-making as concerns raised by students [24].

With regard to the impact of clinical learning on students, the responses gathered indicated that effective clinical teaching and learning accorded them the opportunity to develop competence and confidence in clinical procedures. The clinical environment is the place that allows students to practice the theory learned in the classroom. The current findings agree with a study by Jonsén, Melender, and Hilli [17]. They emphasised the unique role of the clinical environment in the acquisition of skills needed to transition from a nursing student to a registered nurse. The consistent findings could mean that irrespective of where nursing education is pursued, clinical teaching and learning are a common importance.

The general students' perception of clinical teaching was that clinical practice is beneficial; however, most of them believed that there were gaps in theory and clinical practice experiences. The gap in theory and clinical practice could be attributed to the controversy between the ideal versus reality situations in the clinical environment. Most of the items required for demonstrations may be available in the school or classroom. Students go into the clinical environment expecting the same items and equipment. However, in the wards, the situation is different. Most of this equipment is not available, hence the need for improvision. The unavailability of resources mostly leaves these students frustrated and less interested. Previous work reported that core nursing skills learned through modelling tend to achieve higher impact compared with pre-learning levels [25].

Additionally, building competence in core nursing skills will enhance adaptability in the clinical area [20]. The students highlighted some challenges in the clinical setting, primarily referring to the environment not being conducive for learning. In particular, these elements include the following: poor staff attitude, students' complaint that staff nurses were not receptive, shouted at them at least provocation, and others were unwilling to teach or correct them. Contrary to this finding, Antohe and colleagues (2016) purported that students had their staff nurses being the most important professional role model for them. The difference in findings could be attributed to the workload. In developed countries, the nurse-to-patient ratio is lower compared with developing countries. This makes it possible for the staff nurses in the developed countries to have enough time for the student nurses.

In addition, the results of the study found another challenge to be the lack of equipment. Most of the participants revealed that medical supplies and equipment were inadequate; hence, they could not practice much because their supervisors would always remind them not to waste resources. This finding has been reported by Moyimane, Matlala, and Kekana [26]. In that study, they stated that due to a lack of medical equipment, clinical teachers often improvised during clinical teaching; as such, student nurses were not taught ideal clinical scenarios. This explains that the shortage of medical supplies is a common challenge in developing and developed countries.

Moreover, the students found another challenge to be poor student attitude. In this study, students admitted that some of their colleagues had a poor attitude to nursing. They seem less interested in their own clinical learning. Their general motivation in clinical learning was low, and most of them resorted to fidgeting with their phones while on clinical placement. Conversely, the findings of Riklikienė and Nalivaikienė [27] seem otherwise. In their study, student nurses estimated their individual input into clinical training mostly as high or very high. The correlation analysis revealed a positive relationship between the students' assessment of the learning environment in the unit and their individual input to their clinical placement. The different results could be attributed to the lost passion for the profession. In Ghana, some people venture into nursing as a means of livelihood. It is believed that once one completes nursing, there is a ready job available; hence, some people enter nursing school with no genuine passion but as a means to an end.

Lack of learning opportunity was another challenge that was highlighted. Most of the students indicated that they could not have the chance to try out some tasks. This was mainly due to an overwhelming student number and inadequate time that is needed to acquire the needed skills. The findings in this study are inconsistent with a study by AstaMažionienė, Staniulienė, and Gerikienė [28], where they found out that there were enough training cases and situations during their clinical placement. What could account for the difference could be the number of students admitted into the large nursing school and the availability of hospital facilities for clinical training. Most of the nursing schools in developed countries have strict compliance with the number of students to admit. Whereas the situation in Ghana is the opposite, there is a large student population.

Finally, the study found a lack of supervision as another challenge in clinical learning. A supervisory relationship was the most important factor contributing to clinical learning experiences [29]. However, most students complained of inadequate supervision. Students complained of being used as errand boys and girls because there is no assigned supervisor to monitor their work and progress. Most of these students tend to engage in nonprofitable activities making the clinical learning period of ineffective. The results agree with Antohe et al. [30], which revealed the importance of a good supervisory relationship and how it positively influences students' clinical learning experience.

In improving clinical teaching and learning, the participants suggested strengthening supervision. When students know they are being watched, they always do the right thing. Moreover, they suggested that the clinical staff responsible for students should be engaged in rigorous training and workshops. In addition, the students suggest different times for classroom learning and clinical learning, which implies having a specified number of weeks for the theory class and then a different set of weeks for only clinical sessions.

## 5. Strengths and Limitations of the Study

The study design allowed for the exploration of students' perspectives of clinical teaching and learning as it pertains to a bachelors' programme in nursing, which is critical in improving students' clinical nursing practice experiences. The use of telephone interviews did not allow nonverbal cues or body language of the participants to be explored to augment the information they provided. The researchers also acknowledged that the study may be limited by possible recall bias. The participants were expected to share experiences that may have occurred more than six months due to the suspension of clinical learning and teaching activities at the time of interviewing due to the COVID-19 pandemic.

## 6. Conclusions

This study explored nursing students' perception of clinical teaching and learning in Ghana. Although students found the clinical learning environment to be conducive for learning, they encountered challenges that required a holistic approach to address through the efforts of all stakeholders, including students, supervisors, healthcare agencies, and staff. Clinical teaching and learning are critical in facilitating the achievement of clinical competence. The findings suggest the need for innovations in current approaches to clinical teaching to enable students to have autonomy and to be afforded the opportunities to develop essential competencies.

## Figures and Tables

**Table 1:** Demographic characteristics of the participants.

No.	Gender	Age	Programme
1.	Male	21	BSc. Nursing
2.	Female	23	BSc. Nursing
3.	Female	23	BSc. Nursing
4.	Male	25	BSc. Nursing
5.	Female	23	BSc. Nursing
6.	Female	24	BSc. Nursing
7.	Female	24	BSc. Nursing
8.	Female	25	BSc. Nursing
9.	Male	25	BSc. Nursing
10.	Female	22	BSc. Nursing
11.	Male	25	BSc. Nursing
12.	Female	22	BSc. Nursing
13.	Male	24	BSc. Nursing
14.	Female	22	BSc. Nursing
15.	Female	24	BSc. Nursing
16.	Female	23	BSc. Nursing

**Table 2:** Themes and subthemes.

Main themes	Subthemes
(1) Factors that promote clinical learning	(a) Being taught new things (b) Being supervised (c) Having autonomy

(2) The impact of clinical experience on students	(a) Clinical learning opportunity (b) Developing clinical competence

(3) Students' perception of clinical teaching	(a) Clinical practice is good (b) Gaps in clinical practice (c) Inadequate clinical practice

(4) Challenges in the clinical setting	(a) Poor staff attitude (b) Lack of equipment (c) Poor student attitude (d) Lack of learning opportunities (e) Lack of supervisors

(5) Improving clinical teaching and learning	(a) Supervision (b) Training for staff (c) Separate practical and theory sessions (d) Strengthening clinical teaching

## Data Availability

The datasets used and/or analysed during this study are available from the corresponding author on reasonable request.
